# Culturally adapting family interventions for people with schizophrenia in Indonesia: An intervention development study using programme theory

**DOI:** 10.1016/j.ijnsa.2025.100409

**Published:** 2025-08-12

**Authors:** Laoise Renwick, Herni Susanti, Budi-anna Keliat, Dewi Wulandari, Suherman, Rizqy Fadilah, Raphita Diorarta, Helen Brooks, Penny Bee, Karina Lovell

**Affiliations:** a Division of Nursing, Midwifery and Social Work, Faculty of Medicine, Biology and Health, University of Manchester, UK; b Faculty of Nursing, Universitas Indonesia, Indonesia

**Keywords:** Schizophrenia, psychosis, psychosocial intervention, developing countries, complex intervention, programme theory, theory of change

## Abstract

Schizophrenia is a leading cause of disability and global burden worldwide. Low- and middle-income settings are disproportionately affected, and evidence-based, psychosocial interventions are largely unavailable, undeveloped and under-researched for this population. Family interventions have a particularly strong evidence base for reducing relapse risk with medium to large effect sizes. World Health Organisation international clinical guidelines, recommend family interventions as an essential intervention in these settings. Despite this, evidence is emerging that family interventions can deliver positive outcomes in these settings and there are few studies examining the suitability and effectiveness of delivering these in settings with varied socio-economic climates and cultural differences.

We used the Medical Research Council Framework for Developing Complex Interventions, conducting a series of separate but interrelated studies to adapt a testable, evidence-based, culturally relevant family intervention for people with schizophrenia and their families in Indonesia. Our theory driven approach utilises an existing heuristic model to explore stakeholder (service-users, caregivers and healthcare professionals; n = 51) preferences and priorities about family interventions and qualitative interviews with key informants (n = 14) exploring implementation related facilitators and barriers that affect the utility and ecological validity of these interventions. We used a modified nominal group technique to allow divergent ideas to be expressed and collated identifying areas of consensus regarding the optimal content, format and delivery of family interventions.

Our theory of change outlines that while family interventions primarily target relapse, they may also influence broader outcomes like quality of life, functioning, and social inclusion, though not solely responsible for changes in these areas. Therapist confidence depends on training, supervision, and understanding family needs. Intervention components, based on mechanisms of change and stakeholder preferences, align with empirical and theoretical evidence including psychoeducation, communication skills, and relapse prevention. Addressing maladaptive beliefs about schizophrenia, such as hopelessness and blame, was theorised to foster recovery and reduce stigma, a key stakeholder concern.

This theory of change will aid the design of our evaluation, and guide local implementation strategies, inform the development of data collection protocols, and provide a framework for interpreting results. This provides an opportunity to engage stakeholders actively incorporating their perspectives and knowledge into the planning process of the intervention and its implementation, ensuring engagement with policy makers to secure knowledge mobilisation, buy-in and partnership working.

**What is already known**

• Schizophrenia is a leading cause of disability and global burden worldwide.
• Family interventions are known to be effective for reducing relapse risk and improving caregiver outcomes but these interventions are largely unavailable, undeveloped and under-researched in low resource settings.
• Theory-driven approaches are now recommended for developing complex interventions, but few evaluations use programme theory to conceptualise the implementation contexts that influence intervention effects.

Alt-text: Unlabelled box

**What this paper adds**

• We provide an example of programme theory that extends traditional effectiveness/efficacy perspectives to conceptualise the active components of family interventions for schizophrenia and theorise their impact in a new context.
• Our theory of change will be used as a tool to engage stakeholders and promote shared understanding of our intervention and its implementation for further feasibility and efficacy testing.

Alt-text: Unlabelled box

## 1 Background

Schizophrenia is a severe mental illness that affects less than 1 % of the population (Solmi et al., 2023). It is a high burden illness which can have a markedly negative impact on individual social connections, occupational and role functioning and compromise individual ability to maintain independence (Molstrom et al., 2022). Low- and middle-income countries (LMICs) are disproportionately affected, due to a confluence of factors including lack of infrastructure, finite financing and resourcing of services, the availability of adequately trained staff, and low recognition of the need for professional help (Patel et al., 2018). In LMICs, the majority of individuals with schizophrenia do not receive evidence-based, psychosocial interventions and these are largely unavailable, undeveloped and under-researched for this population (Maddock et al., 2021). There is a notable absence of human and healthcare resources, where the average ratio of mental health professionals to patients is as low as 1:200,000 (World Health Organization, 2021). More importantly, many existing healthcare professionals are not equipped with the skills and knowledge to administer psychosocial interventions. Consequently, responsibility for the care of people with schizophrenia falls to their families (Rathod et al., 2017).

It is generally accepted that a narrow package of care focused on medication and brief psychosocial interventions delivered through a collaborative care model are more feasible in lower resource settings due to financial and structural barriers to scale-up and within this, psychosocial family interventions should be prioritised (de Jesus Mari et al., 2009; Jamison et al., 2017; Patel, 2016). Several national and international clinical guidelines recommend the active involvement and support of family members in the care of someone with schizophrenia to harness their caring capacity and capability (Dixon et al., 2010; National Institute for Health and Care Excellence, 2014). The type of family interventions offered should be based on collaborative decision-making among the patient, family and clinician (Dixon et al., 2010) and comprise supportive, educational and treatment functions (National Institute for Health and Care Excellence, 2014). The evidence to support family interventions which are recommended in national guidelines is extensive. Meta analytic studies synthesising evidence from more than 90 randomised trials shows family interventions of several different types are effective for improving outcomes (Rodolico et al., 2022) and when compared with other types of psychosocial interventions that do not focus on involving the family, they continue to show effectiveness and are superior in improving outcomes (Bighelli et al., 2021). Family interventions improve service-user outcomes including functioning (Bighelli et al., 2021), reduce the risk of relapse (Claxton et al., 2017; Pharoah et al., 2010; Rodolico et al., 2022), improve the family environment, adherence to medication and therapeutic alliance between therapist and families (Claxton et al., 2017; Ma et al., 2018; Pharoah et al., 2006; Rodolico et al., 2022). Moreover, these types of interventions are also effective in improving caregiver outcomes including their experience of social support and burden (Lobban et al., 2013).

In low-resource settings, evidence is emerging that family interventions can deliver positive outcomes for individuals with schizophrenia and their families (Morillo et al., 2022). However, the utility and relevance of interventions based on Western concepts and principles has been questioned. Appraisals of schizophrenia, explanatory models of mental illness, cultural beliefs about family, and cultural expression of emotion differ across settings (Degnan et al., 2017; Lim, 2016; Nuraini et al., 2020; Suryani, 2015) The systematic adaptation of interventions transferred from Western settings may thus be warranted to achieve equivalent effects and produce interventions that are acceptable to people in low resource settings (Degnan et al., 2017). Guidance documents have been published which detail intervention development design and processes to enhance the efficacy and function of complex interventions including frameworks to support culturally adapting evidence-based interventions (Skivington et al., 2021). However, intervention development processes are predominantly under-reported, or reported briefly in the context of feasibility studies, which limits understanding of the conceptual and empirical basis for interventions, hampers efforts to model causal mechanisms and relationships between intervention ingredients and outcomes and limits efforts to share learning. Intervention development knowledge gaps can contribute to research waste that arises from inadequate reporting and poor intervention description (Ioannidis et al., 2014) alongside avoidable weaknesses in research design and conduct (Chalmers et al., 2014) which in turn increases the chances that patients may be exposed to ineffective interventions (Bleijenberg et al., 2018).

A key methodology used to optimise the acceptability and feasibility of an intervention is grounding its development in the perspectives and psychosocial context of the people who use them and those who deliver them (Yardley et al., 2015). It is argued that adopting a user-centred approach consisting of an inter-disciplinary research team working together with experts and intervention providers can enhance intervention feasibility, efficacy and effectiveness (Bleijenberg et al., 2018). Integrating user experience into the design of interventions positively impacts on treatment delivery and improves the experiences of patients (Robert, 2013) and engaging stakeholders at an early development stage can yield solutions with greater salience and application in real-world settings (Skivington et al., 2021). However, the feasibility of interventions is also impacted by wider contextual factors, such as socio-demographic factors, logistical issues (Brooke-Sumner et al., 2015) and capacity among healthcare services to deliver evidence-based treatments (Barbui et al., 2020), that are less frequently investigated (Brooke-Sumner et al., 2015). We report the cultural adaptation and refinement of an existing evidence-based intervention for families of people with schizophrenia incorporating a combination of intervention development processes centred on partnering with users and stakeholders and combining theory with prospective implementation assessments (O’Cathain et al., 2019). We aimed to culturally adapt an evidence-based intervention to support families of individuals with schizophrenia using mixed methods incorporating stakeholder views and priorities. We also aimed to develop understanding of the factors that hinder and facilitate psychosocial intervention delivery including training support to enhance the acceptability, sustainability, and implementation of our co-adapted, evidence-based family intervention. We also developed a standardised protocol and training manual detailing how the intervention should be delivered to ensure consistency and fidelity in application across different settings.

### 1.1 Empirical and theoretical basis for family interventions

Family interventions place emphasis on the role of environmental factors, particularly social environment and interactions, that exacerbate symptoms and precipitate subsequent relapse. Family dynamics and patterns of communication are important determinants of relapse (Butzlaff and Hooley, 1998) supporting assumptions that positive and supportive environments are protective while emotionally charged environments over-stimulate and increase propensity for relapse (Barrowclough and Tarrier, 1992). However, knowledge of the exact mechanism(s) of action and the critical elements of family interventions is unclear. Altering family appraisals of symptoms and problems, changing how families understand and respond to symptoms while simultaneously increasing coping capacity through effective problem solving is considered a key process (Barrowclough and Tarrier, 1992). Attributional biases are thought to mediate the relationships between family emotional responses and relapse hypothesising that higher anxiety, depression and lower self-esteem in service-users are the main products of this process (Garety et al., 2008; Kuipers, 2006) that serve to maintain symptoms over time (Kuipers, 2006). As a result, psychoeducational and cognitive behavioural approaches to condition management are favoured in the application of emotional attribution theories to intervention development.

Family interventions are typically complex with individual sessions structured around the individual educational, behavioural and skills-based components. They can be delivered in either multi-family groups or as discrete individual interventions with single families. Evidence does not so far unequivocally support one format over the other (Pharoah et al., 2010) though higher levels of attrition from trials of multifamily groups indicate single family interventions are preferred by those who receive the intervention (National Institute for Health and Care Excellence, 2014). Evidence supports delivering a longer duration intervention (> 5 sessions) as this is associated with better effects on relapse risk reduction (National Institute for Health and Care Excellence, 2014). Best practice guidelines advises intervention duration minimum 3 months (> 10 sessions) and supports delivering the intervention involving the person with schizophrenia in some components (National Institute for Health and Care Excellence, 2014). Family interventions fundamentally share key components in content, focused on information sharing, psychoeducation and needs assessment and also behavioural approaches delivering more complex skills training including coping skills, communication skills, problem solving and goal-setting (Grácio et al., 2016; Harvey, 2018). These essential elements comprising psychoeducation and behavioural skills training are key ingredients of effective interventions in a large meta-anlaysis (Bighelli et al., 2021).

### 1.2 Intervention selection

There are few robust evaluations of culturally adapted interventions that we can draw on to support intervention selection in low resource settings, thus we used England’s national clinical best practice guidelines as the foundation given the wider focus on evidence-based intervention components (National Institute for Health and Care Excellence, 2014). Guidance recommends that interventions are carried out for at least 10 sessions for a minimum of three months and comprise specific supportive, educational and treatment functions including problem solving and crisis management. Elements which can be negotiated include whether it is appropriate to include the person with psychosis in the intervention or whether either single-family intervention or multi-family group intervention are preferable. We were mindful of reviewed evidence from process evaluations and trials that aimed to expound key ingredients and identified common factors valued by intervention recipients including therapeutic alliance, support and the opportunity for sharing (Grácio et al., 2016). We drew on an existing evidence-based intervention primarily underpinned by the principles of cognitive behavioural therapy. These types of interventions can easily be tailored to individual family needs and are endorsed by clinical guidelines (National Institute for Health and Care Excellence, 2013). We chose a complex intervention also because the provision of a comprehensive intervention would allow a broader set of components from which service-users can draw experience and may subsequently allow researchers to isolate the key elements of the process of family interventions that are favoured by participants and potentially those that are related to improved outcomes.

On this latter point, a broader evidence base is required in lower resource settings where there is less evidence to support the implementation of these interventions than there is in higher income settings. Identifying the optimal intervention components will be particularly salient for producing effective and sustainable interventions where there are substantial resourcing challenges. Evidence shows there are some similarities in caregiver burden and emotional strain experienced by Indonesian families of people with schizophrenia and those in Western cultures (Annisa, 2016; Annisa et al., 2015; Tristiana et al., 2018) which suggests that intervention content may be aligned with cultural experiences of illness. However, there are differences in appraisals of schizophrenia, cultural beliefs and explanatory models of mental illness across different settings (Degnan et al., 2017; Nuraini et al., 2020; Suryani, 2015) and differences in cultural expression of emotion (Lim, 2016) which may affect the application of family interventions to these settings. Illness perceptions and views about the utility and effectiveness of treatments have a role in determining the perceived need and acceptability of interventions for individuals in receipt of treatment but also among those who deliver it (Sekhon et al., 2017). Our intervention is based on the model designed by Barrowclough and Tarrier (Barrowclough and Tarrier, 1992) sharing process and delivery features of the model devised by Falloon (Falloon, 2015; Falloon et al., 1993). The intervention comprises separate modules entailing psychoeducation, information giving (feedback), stress management, problem solving and coping skills and communication training. The model is underpinned by cognitive behavioural approaches and founded on stress-vulnerability theories of coping and symptom exacerbation. The intervention supports an individualised approach based on an extended assessment at initial outset to build therapeutic alliances and promotes generalisation of learned skills across situations.

## 2 Methods

### 2.1 Family Intervention for schizophrenia: adaptation and development

**Fig. 1:**
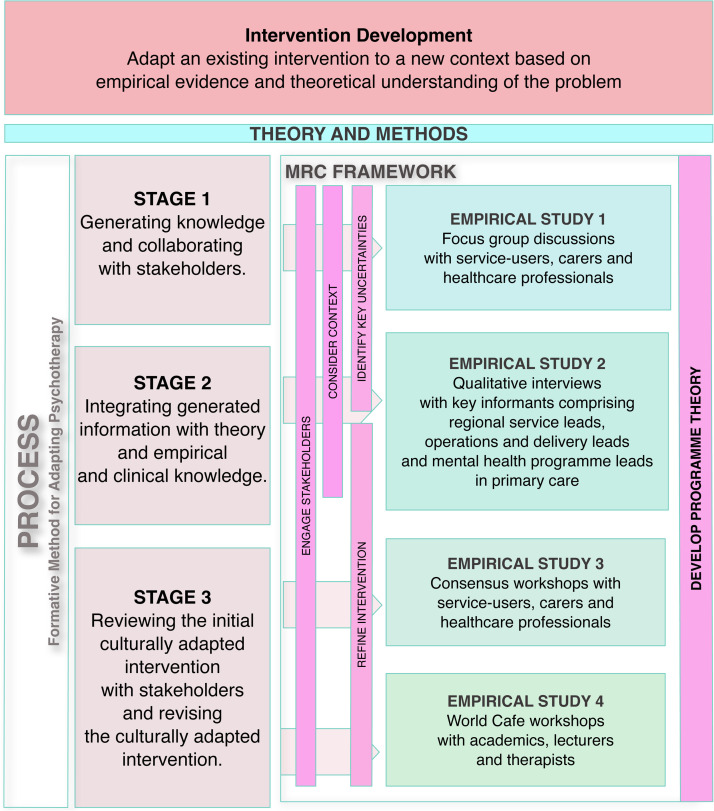
Intervention Development Theory and Methods.

Using the MRC Guidance for Developing Interventions, focusing on the development phase, we conducted a series of separate but interrelated studies to produce a testable, evidence-based, culturally relevant family intervention for people with schizophrenia and their families in Indonesia. We explored contextual features of the intervention to inform further development, evaluation and implementation (see Fig. 1). The process of culturally adapting the intervention was guided by the Formative Method for Adapting Psychotherapy framework which is a community-based, bottom-up approach for culturally adapting evidence based psychotherapeutic interventions (Hwang, 2009). The Formative Method for Adapting Psychotherapy framework guidance comprises 5 stages including 1 generating knowledge and collaborating with stakeholders 2 integrating generated information with theory and empirical and clinical knowledge, and 3 viewing the initial culturally adapted clinical intervention with stakeholders and revising the culturally adapted intervention. The remaining two stages incorporate testing the culturally adapted intervention and finalizing the intervention though we do not report these data here. We focused on intervention adaptation using the first three stages which comprised information gathering, developing a rationale for adaptation and local consultation to verify components of the intervention (Perera et al., 2020) and report testing and finalising the intervention in a separate study. We used a theory and evidence-driven framework informed by the principles of person-based approaches (Alicia et al., 2019), using systematic investigation of stakeholder’s views and needs to enhance the acceptability and feasibility of the intervention during the early stages of development. Intervention adaptation was also informed by an evidence-based, heuristic model for adaptation of interventions for those with schizophrenia comprising content and format for delivery (Degnan et al., 2017). Concurrently, we investigated contextual factors that may affect recruitment and implementation of our prototype intervention to support task-shifting and inform intervention design and delivery (Wight et al., 2016). We adopted an iterative development approach because of the reciprocal relation between the elements in the development phase (Bleijenberg et al., 2018; Skivington et al., 2021) and aimed to produce a manual to accompany our prototype intervention, detailing how the intervention should be delivered (Hoddinott, 2015; Moore et al., 2021). We also held a structured, participative event to bring together different individuals involved in teaching or delivering psychosocial interventions to develop relevant and effective, contextually sensitive training materials to support our manual.

Based on our formative research, we designed an acceptable, feasible and sustainable prototype intervention which was subsequently examined to produce programme theory to guide future refinement, feasibility and effectiveness testing. The map depicts theory of how and why the intervention is perceived to effect change including short and long-term outcomes required to achieve the outcome defined as reduction in relapse risk. The theory details interventions identified in achieving outcomes, the assumptions and conditions that must be met to support progression in the causal pathway, the rationale for interventions and indicators of outcome which can be reasonably perceived to signal achievement of each outcome. The resulting programme theory defines the mechanisms through which the intervention is expected to achieve its effect comprising theory of change and emergent theory of action (Fig. 3). Work on this intervention was undertaken between September 2021 and September 2023 and the subsequent programme theory was developed by the research team in a series of meetings explaining the processes and influences explaining the potential mechanisms of effect to guide future evaluation as each assumption may be tested through feasibility and effectiveness testing (Breuer et al., 2016). The study was granted ethical approval from the University of Manchester Ethical Review Committee (Ref: 2020-8041-13687) and Universitas Indonesia Ethics Committee (Ref: 162/UN2.F12.D1.2.1/PPM2021). Findings are reported using guidance for reporting intervention description (Hoffmann et al., 2014) and informed by guidelines for reporting development studies in health research (GUIDED) (Duncan et al., 2020).

### 2.2 Stage 1: generating knowledge and collaborating with stakeholders

Knowledge generation comprised qualitative consultation groups with a wide range of stakeholders and individual interviews with key informants involved in the planning, organisation and delivery of healthcare in regional sites in Jakarta and Bogor to understand the context and current practice for family involvement in the care of people with schizophrenia.

### 2.3 Interviews with service-users, family members and healthcare professionals

We explored stakeholder preferences and priorities for delivering and receiving family interviews in consultation groups purposively sampling from service user, carer and healthcare professional groups. We conducted two consultation groups per stakeholder group (6 consultation groups with 51 service-users, carers and healthcare professionals) in line with evidence that high levels of both code and meaning saturation can be attained with two focus groups [27]. Topic guides were developed and tested by the study team from our prototype intervention comprising detailed descriptions of the key components of family interventions to inform our inquiry. Data from consultation groups were analysed using the framework method [28, 29]. Data were analysed by Indonesian researchers, and a sample of scripts were transcribed and translated, and coding frame agreed with the wider study team at Universitas Indonesia. Recruitment commenced in October 2021 and was completed in December 2021. The findings are reported separately (Susanti et al., 2024). Carer/s or relatives were included if they were living with or spending at least 10 h per week in face-to-face contact with an individual with schizophrenia and assumed a caring role, were over the age of 18, lived in either Jakarta or Bogor and were able to give informed consent. People with schizophrenia were included if they had a diagnosis of schizophrenia or related psychosis which was confirmed by referring clinician, they were currently receiving treatment in a primary care setting, and were also over the age of 18, a resident of Bogor or Jakarta and able to give informed written consent as judged by the referring healthcare worker. Individuals were excluded if they had a drug or alcohol dependence alongside a diagnosis of schizophrenia, according to DSM-V criteria, unstable residential arrangements such that the likelihood of being available for the duration of the research was low. Healthcare professionals were included if they worked in a primary care setting with responsibility for delivering mental health care. We sampled purposively for gender, age and geographical location to ensure maximum variation within the sampled population. Healthcare workers had specific training in mental health and were responsible for delivering the mental health program within their primary care setting. Having knowledge of formal, structured family interventions was not a criterion for eligibility for inclusion. We sampled purposively for gender, age and geographical location to ensure maximum variation within the sampled population.

Findings revealed core themes relating to concepts and illness models, cultural aspects of family functioning and environment, communication, and cultural norms and practices. In addition, families and service-users identified varied preferences and priorities for format and mode of delivery and key constituents of culturally relevant therapeutic targets. Potential barriers to participation included families experience of multiple conflicting stressors and competing demands for time resulting in difficulties prioritising the treatment of individuals with schizophrenia within the family context. The fear of stigma and discrimination formed a prominent disincentive to participation as the burden of schizophrenia was perceived as a source of stigmatisation for the whole family by service-users, family members and healthcare professionals. A specific need was identified to embed recovery-oriented principles and values to ensure partnership working and decision-sharing between service-users, families and healthcare professionals.

### 2.4 Interviews with key informants

We explored the views of key informants regarding factors affecting implementation of the intervention in primary care settings in individual qualitative interviews. This allowed us to understand the wider implications of intervention implementation and evaluate factors affecting reach, adoption and maintenance of interventions devolved to non-specialist mental health workers in primary care settings (Pahwa et al., 2023). Community public health centres or primary health centres (called *puskesmas*) are an important provider of services to people with serious mental illnesses in the community and are tasked with providing a minimum level of mental health care and this was the focus of our inquiry. Key informants are individuals who have in-depth knowledge about the community and the target population, community access for healthcare and processes involved in the distribution and delivery of mental health services, and as such are best positioned to identify barriers and facilitators to implementation of interventions and the successful completion of a feasibility study and future trial.

We used snowball sampling and a two-pronged approach to identify interview participants through leads for mental health programme delivery in our research sites (Bogor and Jakarta) and through our collaborative network workshops with 22 high-level stakeholders from the Ministry of Health, academic organisations and non-governmental organisations held in Manchester in July 2019. We systematically categorised recruited participants as academic, government, organization/association, and healthcare/clinical representatives based on geographical location to ensure equitable representation. We recruited 14 key informants comprising roles such as city-level coordinators of mental health, provincial heads of disease prevention and control and heads of regional and district primary care centres. Interviews were conducted by phone or in-person based on the preference of the respondent and each interview lasted approximately 60 minutes. Interviews explored perceptions of need and mental health service delivery, perceptions of psychosocial therapies and factors that affect the implementation of these in primary care. We explored the feasibility of delivering family interventions in different contexts and for different modes of delivery, potential engagement and recruitment issues, resource availability, current knowledge and experience of family interventions and the degree to which primary care services could facilitate delivery of these interventions by non-specialist health workers. Transcribed interviews were coded inductively using thematic analysis by the lead researcher (Braun and Clarke, 2006) to identify relevant issues for implementation. Emergent themes were developed in consensus with the wider study team to agree the content and composition of themes comprising sub-themes and quotes were selected to characterise each of the themes.

Key informants were concerned that limited resources available for providing talking therapies may hamper delivery. Task-shifting was explored as a potential solution to capacity constraints, enabling lay workers and other non-mental health professional workers e.g. social workers, occupational therapists and general psychologists to deliver the intervention. Local volunteers (*called kaders*) already support mental healthcare delivery providing illness detection, treatment conveyance, and education in their communities. Their role as a trusted intermediary between communities and healthcare professionals placed them in an advantageous position to promote engagement with the intervention though consideration should be given to the perceptions of their role among community members for optimising engagement. Community awareness and engagement could conceivably be enhanced by collaborating with community leaders (village head, regional leaders, religious leaders) who could promote better understanding of the need for treatment among families. This was underscored by perceptions among informants that prioritisation of talk therapies may be influenced by individual beliefs about the effectiveness of treatment, competing demands for time and socio-economic status. Patient and family refusal was viewed as among the greatest foreseeable obstacles to implementing family interventions in practice; refusal is driven by stigma and low perceived need for treatment among families. Socio-economic status of families was considered a specific factor that needed consideration in designing our intervention and tailored approaches may be needed for different socio-economic groups to enable access and content required adaptation based on background education.

### 2.5 Stage 2: integrating generated information with theory and empirical and clinical knowledge

The empirical work described above was summarised in an evidence matrix guided by the methodology of Lovell et al. (Lovell et al., 2008), which allowed the study team to understand commonalities and disagreements between stakeholder views from different sources of evidence to support decision-making regarding possible content, duration and delivery of the intervention (see Table 1). Data from each study were analysed and reported separately using qualitative analytic methods. Synthesised information was integrated with empirical evidence from the wider literature by the first author and discussed with the wider study team in Indonesia comprising professor of mental health nursing, lecturer in mental health nursing and five post-graduate mental health nursing researchers and mental health nurse specialists. Ideas were generated among the team from these data and aligned with practice norms emerging from the wider literature which are highlighted in the textual description of our Theory of Change. There were constraints on the mode and content of delivery of family interventions based on evidence of active ingredients which were discussed. The prototype manual was then reviewed by a family interventions expert (Dr Tim Bradshaw) and further adaptations made to content based on clinical experience. Areas of divergence were taken forward to a consensus workshop in stage 3 using nominal group technique (NGT), to obtain agreement on aspects of intervention delivery that were not clearly agreed following the synthesis in stage 2.

**Table 1:** Intervention Evidence Sources.

	Evidence Source				Included in Adapted Intervention
	*Service-users*	*Caregivers*	*Healthcare Professionals*	*Key Informants*	
*Cultural* *Component*	*-Incorporate explanatory models that include spiritual beliefs and attributions of religion to mental illness* *-Engendering hope in HCPs philosophical approach to treatment key for therapeutic alliance* *-Families central to recovery and interventions should include parents, spouses, and siblings* *-Support families to support service-users* *-Family involvement in care is important* *-Preferred communication with professionals via digital applications i.e. WhatsApp* *-Service-users emphasised the importance of the initial assessment, including families understanding of the illness, their burdens in taking care of family members with mental disorders, how families find solutions for their own problems, how mental illness affect the patients and the families and about the strengths of the family.* *-Service-users were also largely in agreement* *that they should attend some sessions with their families*	*-Needs understood to mean access to material resources necessary for life e.g., food, water, shelter and should reframe psychological needs as ‘problems’* *-Family members prioritised different components* *of FI based on their own experiences although stress management was highlighted among* *multiple narratives from both families and service-users as a specific priority.* *-Shift focus from coercive medication strategies to inclusive and informed decisions with HCPs* *-Attending to spiritual needs were crucial for countering stress, both experienced by families and as a tool to buffer against negative illness effects in service-users*	*-Target misconceptions about symptom attributions and perceived controllability of symptoms* *-Equip HCPs with skills to target disengagement and deal with complex illness presentations/refractoriness* *-Focus on promoting independence to reduce paternalism* *-Target family denial of illness through sustained engagement and tailored psychoeducation* *-Target stigma and misconceptions among HCPs* *-Concerned about involuntary disclosure if delivering the intervention in the patient’s home*	*-Develop educational resources focused on dispelling myths and misunderstanding about mental illness and emphasise the value of talking therapies* *-Engagement with community assets to create pathways to intervention delivery and receipt* *-Enable participation through negotiating with community leaders* *-Consider the possibility of devolving roles in intervention delivery to local volunteers (kaders)*	*-Emphasis of the intervention should be on working with varied explanatory models of illness* *-Include components on spirituality and religion e.g. modified case study* *-Amending terminology around problems vs needs* *-Education emphasising common myths and misconceptions* *-Focus on reduction of paternalism e.g. encouraging patient choice regarding medications*
*Content*	*-Relapse prevention a prioritised intervention and fundamental component* *- Families could* *monitor individuals to detect relapse earlier and facilitate earlier support with the right information and support* *-Families role in planning relapse prevention strategies* *-Minimise homework to reduce caregiver burden*	*-Relapse prevention a prioritised intervention and* *fundamental component* *-Continued medication administration to prevent relapse.* *-Highlighted a priority to focus on relapse prevention* *-Effective collaboration and communication between families and HCPs based on equal partnership* *-Meaningful recovery and attaining independence are key targets* *-Open discussion with HCPs around medication* *-Preferred format for intervention-related resource is written e.g., leaflet, handouts* *-Promote encouragement, support and hope* *-FI should have a specific focus on reducing family* *stress and enhancing wellbeing*	*-Information-giving and problem-solving techniques* *- Relapse prevention was a prioritised intervention and* *fundamental component of family interventions from* *the perspective of service-users, families, and health-* *care professionals.* *- Focus on medicines management for relapse prevention* *-Treatment goals comprise improved knowledge, self-confidence, adherence to medicines, enhanced social support, greater family wellbeing and improved communication*	*-Acknowledge varied explanatory models of illness* *-Adapt intervention, materials and resources to educational background/socio-economic situation of participants* *-Knowledge and information sharing, targeting stigma*	*-Provide simplified explanations of key concepts e.g. stress-vulnerability model replaced by ‘stress bucket’* *-Include specific relapse prevention component e.g. ‘Back in the Saddle’* *-Emphasis on stress management skills and family wellbeing* *-Communications skills are a key component* *-Therapeutic targets focused on social inclusion, supporting patients with return to employment and meaningful activity* *-Positive regard for service-users throughout endorsed by families, HCPs and service-users* *-Training materials emphasise partnership working*
*Process and Format for delivery*	*-Adaptation to setting needed for feasible delivery* *-* U*ndecided about whether the interventions should take place* *at home or in the primary care centre*	*-Preference for delivery of FI at home to preserve privacy but also for pragmatic reasons e.g. travel, time management* *adaptation to setting needed to ensure feasible delivery* *-Broadly agreed on ten sessions based on model presented but suggested workarounds that would facilitate individual* *attendance based on existing schedules and priorities such as conducting the intervention at home or having shorter sessions* *-Collaborative working with HCPs needed* *- Focus on therapeutic and gainful employment to provide opportunities for greater social inclusion*	*-Adaptation to setting needed for feasible delivery* *- Concern about stigma* *and involuntary disclosure if interventions were provided in the family home* *-FI could conceivably be delivered alongside existing programmes already delivered in the community to reduce workload and enhance implementation* *- Concerns about the feasibility of delivering FI that are intensive with consistency, i.e.,* *individual families and multiple sessions with existing workloads, competing demands, staffing resource shortages and a lack of skilled and trained staff to deliver existing programmes* *-Suggested the use of robust therapist training with continuous training throughout to enhance confidence of FI therapists*	*-Flexible appointments to enable engagement and retention* *-Incentivise participation/reimbursing families for taking part in the intervention* *-Reduce intervention components, number and duration of sessions to mitigate against resource constraints* *-FI can feasibly be delivered by primary care workers* *-Uncertain about whether untrained workers could feasibly deliver this intervention, potentially able to deliver discrete components such as psychoeducation* *-Provide FI in the primary care setting or some elements in group format*	-Flexible appointments, offered in families home setting -Delivered as a single-family intervention -FI to be delivered by non-specialist healthcare professionals within primary care -FI therapists to work collaboratively with community leads and volunteers to promote awareness, understanding and support increasing demand for attendance -FI delivered in 10 sessions, approximately 60 minutes per session -Include service-users in some sessions but not all e.g. psychoeducation and feedback

2 - FI = family intervention, HCP = healthcare professional.

### 2.6 Stage 3: reviewing and revising the initial culturally adapted intervention with stakeholders

The final stage before feasibility testing our intervention involved obtaining consensus on further adaptations to the manual, translation and review of the manual by clinicians delivering the intervention. Nominal group technique has been effectively implemented in mental health research (Evans et al., 2017; Tyler et al., 2020) to allow divergent ideas to be expressed and collated with a view to identifying areas of consensus. Each item was presented, voting was conducted anonymously and participants were able to add context to their decision in a free text response that remained anonymous but could be viewed by the entire group to stimulate discussion. Researchers facilitated structured discussion until consensus was reached for items where there was not a clear majority. We recruited stakeholders comprising service-users, carers and healthcare workers utilising the same methods and eligibility criteria as Study 1 but these were participants that had not previously taken part in this study. 22 participants (16F, 6M) attended the consensus workshop comprising family members (n = 7), service-users (n = 7) and healthcare professionals (n = 8). Data were collected in May 2022. Agreement was obtained by majority consensus (> 75 % concordance) on most areas of divergence during initial voting. Respondents believed that sessions should be conducted in the family home, be approximately 60 minutes duration, with the patient attending some elements. The majority of respondents were in favour of sessions including service-users but there were mixed views on multi-family groups. Most (n=13) opposed these and while many respondents could see there was value in this for sharing stories and experiences and supporting each other, there were strong feelings among many that the intervention should be delivered as a single-family intervention. Our manualised intervention was further developed by gathering ideas for training and development of our intervention manual from academics, researchers and intervention developers (n = 22) in a World Café event which took place in July 2022 on the university campus and lasted 120 minutes. Four topics her posed and each group engaged with the discussions at each table for 25 minutes. Training required, additional materials and optimal delivery format were discussed and methods described including the qualities of effective teachers and trainers, case demonstration, problem-based learning and effective resources. The protocol for intervention delivery was improved by refining the methods for training, the inclusion of supporting evidence for each section, teaching methods and optimising the duration and format of training sessions.

## 3 Results

The prototype intervention comprised i) a training manual comprising culturally adapted intervention components (gathering information, providing feedback, communication skills, stress management, problem-solving & coping skills and relapse prevention) separated into workshops and accompanying evidence base and culturally acceptable teaching and learning activities, ii) psychoeducational booklets detailing myths and misconceptions linked with specific family intervention components and iii) a supervision framework to support therapists to deliver family interventions consistently and confidently.

**Fig. 2:**
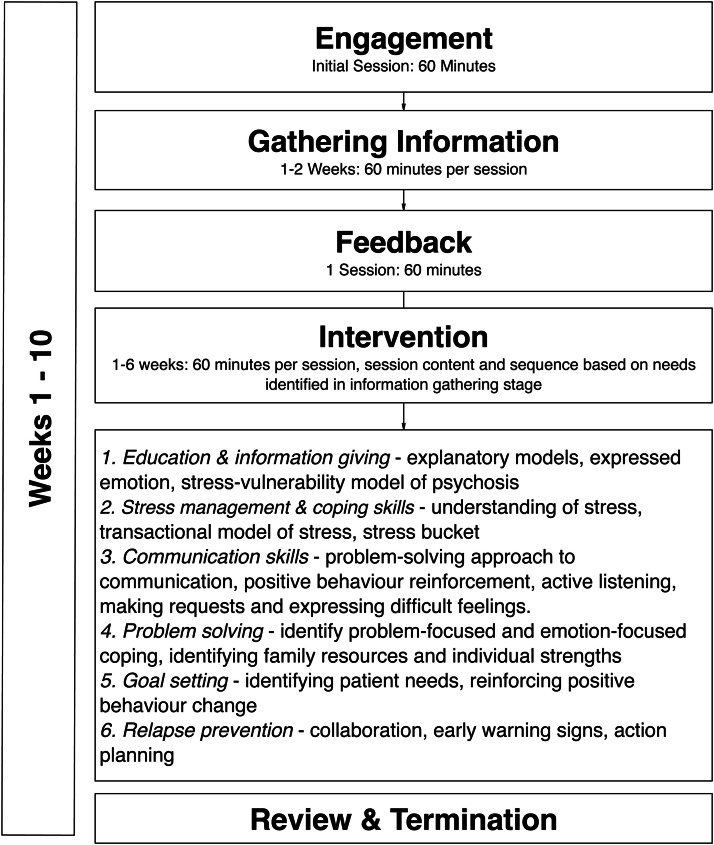
Intervention Content and Process.

Training was delivered using the co-developed training manual to enhance both knowledge and skills in delivering components of family interventions. Training lasted 4.5 days and was co-delivered by UK and Indonesian researchers and mental health nurses led by a second family interventions expert (Ms. Cath Gamble). In addition to training slides for each of the family intervention components listed above, training slides were created detailing the evidence base for family interventions, the philosophical underpinnings, workshops covering the principles and skills of active engagement and cognitive behavioural approaches all of which are covered in the manual. The prototype family intervention consists of 10 sessions, delivered to families of people with schizophrenia (parents, siblings and extended family living in the family residence) including the person with a diagnosis of schizophrenia over a period of three months. The structure and accompanying programme theory are presented in Fig. 2 as the basis for further development and testing of our culturally adapted family intervention in a feasibility trial with nested qualitative process evaluation.

**Fig. 3:**
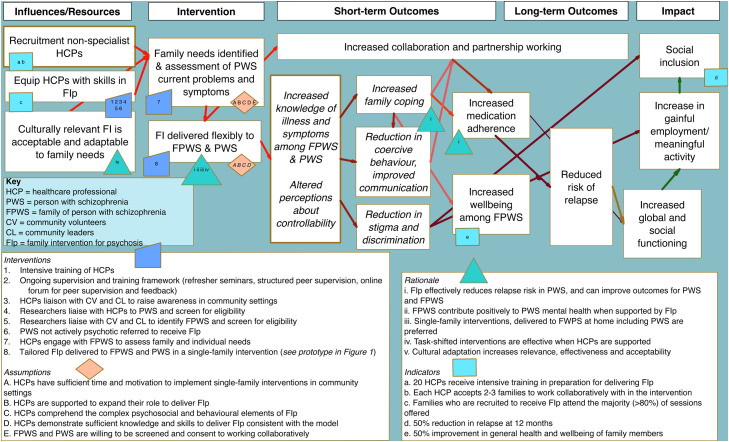
Theory of Change.

### 3.1 Programme theory

The long-term outcome of culturally adapted family interventions was identified a priori as reduction in relapse risk based on existing evidence (Pharoah et al., 2010). The threshold at which culturally relevant family interventions reach the ceiling of accountability, details impacts with a wider sphere of influence within the broader environment, comprising objective quality of life indicators, functioning and social inclusion. Family interventions may contribute to achieving impact in these spheres through impacts on relapse risk but so not solely contribute to these outcomes.

Based on existing published evidence and our empirical work, we identified pre-conditions that need to be fulfilled to ensure successful intervention delivery and feasibility trial. There is substantial evidence that task-shifting is effective for providing cost efficiencies (Seidman and Atun, 2017) and when implemented effectively, can be a valuable strategy for improving healthcare access in resource-constrained settings (Joshi et al., 2014). Task-shifting was cited among key informants as a necessary solution to resource constraints and identifying the availability and willingness of sufficiently skilled non-specialist workers is an essential first step in implementation (Susanti et al., 2024) alongside the availability of these workers to be released from duties to attend for intensive training.

Empirical work informed the culturally relevant intervention, utilising extensive stakeholder engagement and heuristic models of cultural adaptation (Degnan et al., 2018; Rathod et al., 2019), enhancing the acceptability of our intervention. Therapist’s confidence in tailoring the intervention is linked with experience, training, ongoing supervision and knowledge of family needs (Eassom et al., 2014). Intervention components were derived from existing evidence exploring the mechanisms of change in family interventions and contextualised with stakeholder views, and preferences and priorities for delivery. There is strong evidence that family psychoeducation significantly reduces relapse in individuals with schizophrenia by up to 20-50 % compared to standard care (Pharoah et al., 2010).

Psychoeducation increases awareness of illness symptoms, the importance of regular medication and treatment engagement when families are involved in care alongside service-users (Lincoln et al., 2007) thus the foci of our intervention was both families and service-users. Family functioning and communication are improved by focusing on expressed emotion, a known predictor of relapse (Butzlaff and Hooley, 1998). As dysfunctional communication can lead to misunderstanding and conflict, communication skills component focused on helping families to express themselves openly and supportively. Relapse was prioritised among stakeholders to enhance proactive responses and both family and service-users ability to recognise early warning signs (Morriss et al., 2013). Strong evidence from network meta-analysis highlights that psychoeducation, where families are taught problem-solving skills alongside early warning signs recognition, reduces relapse risk significantly (Rodolico et al., 2022). Lastly, addressing maladaptive beliefs about schizophrenia such as hopelessness and blame supports recovery-oriented attitudes and reduces stigma (Vass et al., 2015), which was highly prioritised among stakeholders.

## 4 Discussion

We report on systematic, empirically driven research to design a culturally relevant intervention for families of people with schizophrenia and identify mechanisms of change through which culturally relevant family intervention may lead to real-world change. In response to limitations of the Medical Research Council framework and recognising that part of the complexity in complex interventions arises from the interaction between the family intervention and its context (Skivington et al., 2024; Wallner et al., 2023), we focus on generating a theory that can explain how and why family interventions might work in this context in Indonesia and we will use this theory to guide further feasibility testing and analysis of real-world impact using a pragmatic trial design. We adopted a theory driven approach incorporating theory of change into the Medical Research Council framework and we produced an evidence-based theory revealing causal pathways that influence key mechanisms in the intervention combining empirical evidence, theoretical and heuristic frameworks and wider stakeholder experience and expertise.

Programme theory can improve the initial design of an intervention that is more likely to work within the constraints of available resources within the delivery context and as such, is more likely to improve effectiveness (De Silva et al., 2014; Durlak and DuPre, 2008). For our purposes, the theory of change serves as a framework to identify key factors influencing the success or failure of implementing our intervention and will help to prioritise areas to investigate during our planned feasibility evaluation. Defining the components of an intervention, and how these are expected to cause changes, that contribute to its complexity is vital for methodological transparency enabling research to identify the relative influence of different parts of the intervention on outcomes (Clark, 2013). While there is a small but increasing evidence base showing favourable economic effects of providing family interventions in particular to inform decision making in low and middle income countries (World Bank Group, 2018), there is a need to recognise the varied contexts and organisational, social, cultural and geographical systems that contribute to intervention effects and there are few robust evaluations of family interventions effectiveness and implementation in low resource settings. Family interventions are relatively theoretically advanced regarding the purported mechanisms that lead to individual effect, family functioning and caregiver wellbeing, however, the exact mechanisms of action, active or key ingredients are unknown (Grácio et al., 2016) which hampers advances towards providing efficient, evidence-based treatments.

Importantly, our theory of change may enable identification of parts and processes within our intervention which are essential and those which are potentially expendable, and this is particularly salient in low and middle income countries where resources to support delivery and scale-up are scarce. Recent evidence argues that where there are financial constraints providing psychoeducation alone should be prioritised given its comparative effectiveness against other intervention formats demonstrated in network meta-analysis (Rodolico et al., 2022), however, there remains a paucity of research on family interventions in lower resource settings. More nuanced theoretical exposition of the implementation factors that influence the strength of the relationship between family interventions and relapse may permit analysis of both implementation and intervention effects advancing knowledge of what works as well as for whom and how. Where fewer resources exist to support service delivery, identifying the most suitable resources, tasks and activities needed to support implementation and determining appropriate allocation of those resources takes on greater significance.

While our theory of change is informed by various data sources, we relied heavily on stakeholder experiences and views, incorporating multiple perspectives to generate our theory. We recognise the need to advance our programme theory to further explicate stakeholder’s assumptions and promote shared understanding about how a programme works. Engagement with service users and their families and caregivers is viewed as essential for successful implementation, as this can reduce stigma, increase knowledge and improve attitudes towards people with mental illnesses and treatments (Davies and Lund, 2017; Lavery et al., 2010). Acceptability as a construct is multi-faceted and reflects individual perspectives on whether the intervention is considered useful and appropriate. Effectiveness can be compromised by patient views regarding burden, perceived effectiveness, ethicality, intervention coherence and self-efficacy (Sekhon et al., 2017). Similarly, engaging wider stakeholder groups, leveraging existing trusted relationships early is key to promoting successful engagement among several spheres of influence from policy makers to service planners and managers, non-governmental organizations, service providers, traditional and spiritual leaders, and representatives of other community organisations will optimise the success of implementing interventions in wider contexts (Davies and Lund, 2017; Javadi et al., 2017).

The theory of change will also aid the design of our evaluation, and guide local implementation strategies, inform the development of data collection protocols, and provide a framework for interpreting results. This provides the opportunity to engage stakeholders actively incorporating their perspectives and knowledge into the planning process of the intervention and its implementation, ensuring engagement with key stakeholders including service managers, planners and policy makers to secure knowledge mobilisation, buy-in and partnership working (Keynejad et al., 2016; Lund et al., 2012). Further research should extend scientific enquiry to explore whether culturally adapted interventions work in new contexts, and if so generate understanding of key mechanisms and moderators based on theoretical understanding of isolating active components of interventions and their impact on the intervention (Wallner et al., 2023).

## 5 Conclusions

We have adapted a culturally relevant intervention incorporating programme theory to develop theory-based evaluation processes for further research on family interventions. Family interventions are theoretically advanced and underpinned by robust empirical evidence regarding effectiveness but data to support the implementation of family interventions in low resource settings is minimal and globally, there is scant research which examines the interaction between the intervention itself and contexts in which they are delivered, contributing to an evidence vacuum and significant knowledge gaps about wider factors that influence implementation and adoption of the intervention in newer contexts. Our study fills this gap, applying rigorous methods to the adaptation of these interventions to low resource contexts through extensive stakeholder engagement.

## CRediT authorship contribution statement

**Laoise Renwick:** Writing – review & editing, Writing – original draft, Project administration, Methodology, Investigation, Funding acquisition, Formal analysis, Data curation, Conceptualization. **Herni Susanti:** Writing – review & editing, Writing – original draft, Resources, Project administration, Methodology, Investigation, Funding acquisition, Formal analysis, Data curation, Conceptualization. **Budi-anna Keliat:** Writing – review & editing, Supervision, Resources, Project administration, Methodology, Investigation, Funding acquisition, Formal analysis, Data curation, Conceptualization. **Dewi Wulandari:** Writing – review & editing, Resources, Project administration, Methodology, Investigation, Formal analysis. **Suherman:** Writing – review & editing, Resources, Project administration, Methodology, Investigation, Formal analysis. **Rizqy Fadilah:** Writing – review & editing, Resources, Project administration, Methodology, Investigation, Formal analysis, Data curation. **Raphita Diorarta:** Writing – review & editing, Project administration, Methodology, Investigation, Formal analysis, Data curation. **Helen Brooks:** Writing – review & editing, Methodology, Investigation, Funding acquisition, Formal analysis, Conceptualization. **Penny Bee:** Writing – review & editing, Methodology, Investigation, Funding acquisition, Formal analysis, Conceptualization. **Karina Lovell:** Writing – review & editing, Methodology, Investigation, Funding acquisition, Conceptualization.

## Declaration of competing interest

The authors declare that they have no known competing financial interests or personal relationships that could have appeared to influence the work reported in this paper.

## Data Availability

Participants are identifiable in the qualitative data and sensitive information has been withheld. Deidentified data will be shared in the institutional repository (https://figshare.manchester.ac.uk) following publication.
